# Exploring demands from the perspective of employees identified as being at risk of burnout

**DOI:** 10.1080/17482631.2017.1361783

**Published:** 2017-08-08

**Authors:** Cecile Gauche, Leon T. de Beer, Lizelle Brink

**Affiliations:** ^a^ WorkWell Research Unit, North-West University, Potchefstroom, South Africa

**Keywords:** Job demands, life demands, burnout, health-impairment process

## Abstract

**Purpose**: Burnout has become an occupational health concern. However, little is known about the experiences of individuals identified as being at risk of burnout. This study aimed to address this gap by exploring employees’ experiences of well-being who were identified as burnout risks.

**Method**: Interviews were conducted with 26 employees who agreed to participate in the study. A phenomenological approach was taken, with a case study design as the research strategy.

**Results**: Three major themes were identified: job demands, life demands, and health concerns. It was evident that participants were experiencing demanding conditions in both their work and personal lives, indicating burnout to be a multi-domain phenomenon.

**Conclusions**: Professionals and managers should take note of these results to assist and support employees who are identified as being at risk of burnout.

## Introduction

Employees are a critical resource in enabling organizations to deliver on strategic intent and achieve a sustainable competitive advantage (Wagner & Hollenbeck, 2015). Overall, employee well-being (life evaluation, emotional health, physical health, healthy behaviour, work environment, and basic access) has been found to predict future employer outcomes related to health, productivity, and retention (Sears, Shi, Coberley, & Pope, 2013). It could be argued that this should make the well-being of employees a critical focus area for organizations, yet this is seldom the case. In the current economic climate, employees are often expected to deliver greater outputs with fewer resources, often expressed by the mantra “Do more with less” (Evenstad, 2015). This leads to increased pressure experienced by employees, negatively affecting their well-being into an eventual state of burnout (De Beer, Rothmann Jr., & Pienaar, 2012; Steinhardt, Smith-Jaggars, Faulk, & Gloria, 2011).

Burnout is an occupational concern which negatively impacts both the health of employees and work-related outcomes in organizations (Bakker, Demerouti, & Sanz-Vergel, 2014). Burnout can be described as a psychological syndrome characterized by exhaustion, cynicism, and reduced professional efficacy (Maslach & Leiter, 1997). Anand and Arora (2009) described employees who are suffering from burnout as being emotionally exhausted, detached from their clients and work (cynicism/depersonalization), and feeling unable to achieve their goals (professional inefficacy). There is strong empirical evidence implicating inordinate job demands as an important predictor of burnout (Alarcon, 2011; De Beer, Pienaar, & Rothmann Jr., 2016; Lee & Ashforth, 1996).

According to Bakker and Demerouti (2007), the job demands–resources (JD-R) model is a theoretical framework that can be used to explain not only the impact of job demands, but also the impact of job resources on work-related well-being (consisting of burnout and work engagement), and comprises two processes: a health-impairment process and a motivational process (Schaufeli & Bakker, 2004). While the motivational process indicates that job resources lead to organizational commitment via work engagement (De Beer et al., 2012; Schaufel & Bakker, 2004), the health-impairment process holds that high job demands (e.g., workload and emotional demands) drain employees’ mental and physical resources, leading to the depletion of energy (Bakker & Demerouti, 2007), and also to psychological and physical health problems (Bakker et al., 2014; De Beer et al., 2016). For the purposes of the current study, the research focused on the health-impairment process of the JD-R model as a foundation to explore demanding aspects of employees’ lives.

When investigating demands experienced by employees, it is important to consider both job demands and life demands (Bianchi, 2016). Dyrbye et al. (2006) confirmed that despite the notion that burnout is mainly linked to work-related stress, a strong relationship also exists between personal life events and professional burnout—highlighting the importance of investigating both job and personal demands in creating a better understanding of burnout as experienced by employees. The impact of job demands on burnout has been explored by various researchers (e.g., Alarcon, 2011; Seidler et al., 2014), and typically include aspects such as work overload, emotional demands, mental demands, work–home interference, role ambiguity, role conflict, role stress, stressful events, and time pressure (Olivares-Faúndez, Gil-Monte, Mena, Jélvez-Wilke, & Figueiredo-Ferraz, 2014; Lee & Ashforth, 1996; Peeters, Montgomery, Bakker, & Schaufeli, 2005; Schaufeli, Bakker, & Van Rhenen, 2009). In investigating the impact of life demands on burnout, life events have been found to have an impact on burnout risk, e.g., divorce, personal illness, illness or death of a close family member, marriage, birth, and adoption of a child (Dybre et al., 2006).

Consequently, burnout has been linked in the literature to both psychological and physical ill-health symptoms. The relationship between burnout and depression is frequently explored in the literature (Bianchi, Schonfeld, & Laurent, 2015); burnout has been reported to predict depressive symptoms (Ahola, 2007) and life dissatisfaction. Thuynsma and De Beer (2016) also found depressive symptoms and satisfaction with life (together with job demands) to explain significant amounts of variance in the burnout construct. Burnout has been demonstrated to also lead to poor physical health, including sleep disturbances, headaches, respiratory infections, and gastrointestinal infections (Kim, Ji, & Kao, 2011), as well as musculoskeletal problems (Armon, Melamed, Shirom, & Shapira, 2010). Kivimäki and Kawachi (2015) reported in a review of evidence from 27 cohort studies, of more than 600,000 individuals, that work stressors such as job strain and long working hours are associated with a moderately elevated risk of incident coronary heart disease and stroke. The same study also showed an association between work stress and type 2 diabetes (Kvimäki & Kawachi, 2015). Research in South Africa has shown that burnout is linked to self-reported treatment for diabetes, hypertension, and irritable bowel syndrome (De Beer et al., 2016). Therefore, it is clear that there is a link between burnout and health.

Although previous studies have produced a long list of possible predictors of burnout, a full understanding of the process and experience of burnout is still lacking (Ten Brummelhuis, Ter Hoeven, Bakker, & Peper, 2011). In a qualitative study aimed at obtaining a more in-depth understanding of how nurses experience long-lasting stress and burnout, changes and reorganizations were highlighted by participants as stressors (Billeter-Koponen & Fredén, 2005); the process of decision-making where reorganization occurred without consideration for the knowledge and experience of nurses, despite the fact that decisions have a significant impact on their daily work, was specifically pointed out. Athlete burnout was also explored qualitatively by Gustafsson, Hassmén, Kenttä, and Johansson (2008), who found athlete burnout to be a complex interaction of multiple stressors (e.g., “too much sport”), inadequate recovery, and frustration from unfulfilled expectations. A qualitative research approach is beneficial as it allows deeper insight into the issues being studied (Patton, 2002) from the perspective of employees, as was also the case in the current study.

### Research purpose and objectives

There is a need for integrative research in the field of industrial and organizational psychology to combat the risk of unreliable results associated with continued compartmentalization of occupational and non-occupational domains in research on burnout (Bianchi, 2016). This qualitative study set out to explore the demanding aspects (both job demands and personal demands) of the lives of employees who identified as being at risk of burnout. Having a more comprehensive understanding of the experience of burnout by employees, and the role of job and personal demands, will enable organizations to better support employees, contribute to the creation of an environment that will allow employees to function optimally, and simultaneously contribute to the field of organizational research.

## Method

The study was grounded within the social constructivism paradigm. The social constructivist worldview considers the specific contexts in which people live and work, and the aim of the research is to be guided as much as possible by the participants’ views of the situation being studied (Creswell, 2013). Through interactions between the researcher and individuals being studied, the researcher made sense of the views that others constructed within their minds, while being aware of and acknowledging their personal, cultural, and historical experiences (Creswell, 2013)—this is the essence of constructivism.

A phenomenological approach contributed to the understanding of everyday experiences of participants and allowed the researcher to gain deep insight into the manifestations of reality (De Vos, Strydom, Fouché, & Delport, 2005) while refraining from placing emphasis on a preconceived idea of the phenomenon (Converse, 2012). This approach allowed the study to reflect an accurate and authentic description of experiences and views as shared by the participants, and allowed the researcher to gain deeper insight and understanding into the way that employees identified as being at risk of burnout experienced their demands.

As a research strategy, a case study design was employed. A single case study strategy was used, in which the organization represented the unit of analysis where the phenomenon of burnout was studied. In this study, the researcher aimed to explore the experience of demands from the perspectives of employees identified as being at risk of burnout within the organization.

### Participants and sampling

The research was conducted within a specific business unit/division of a financial services organization based in South Africa. The financial organization functions with a decentralized operating model, i.e., every business unit aligns its strategy to the overall business strategy and hence determines its own people management strategy. The particular business unit had departments ranging across various functions including multiple call centres (e.g., sales, client services, and claims) as well as organizational support departments such as finance, actuarial, information technology, and human resources. Roles in the business unit ranged from operational positions (e.g., call centre advisers) to senior management. The particular business unit had a headcount of approximately 300 employees.

A combination of purposive and convenience sampling was used in the present study. The criterion used to select participants in the study was employees from the population group referred to above who were identified as being at risk of burnout through the use of the annual organizational climate survey. The climate survey (Organisational Human Factor Benchmark) included a burnout module from the South African Employee Health and Wellness Survey, which has been validated for the South African context (e.g., De Beer et al., 2012). The items form a total score for burnout (Cronbach’s alpha coefficient for implementation was 0.89; acceptable) based on its core components (exhaustion and cynicism) (De Beer & Bianchi, 2017), which was then compared against a norm of more than 50,000 employees in the South African workforce. Those who scored above the norm were considered as potential participants. In total, 49 employees were identified as being at risk of burnout and 26 employees agreed to participate in an interview. Participants completed a biographical questionnaire which provided us with the following information. Of all the participants, 14 (54%) were female and 12 (46%) male. The largest proportion of participants were White people (*n *= 13; 50%), while seven (27%) were Black African people, five (19%) were Indian people, and one 4% was a Coloured person.^1^1.This is an official and accepted term in South Africa, designating citizens of mixed ethnic origin. The mean age of the participant group was 31.08 years (SD = 5.94); specifically, the majority of participants were between the ages of 25 and 35 years (*n *= 17; 65%), while three (12%) were younger than 25 years, and six (23%) were between the ages of 35 and 45 years.

### Data collection

Semi-structured interviews were used to explore the experiences of work-related well-being from the point of view of participants. Interviews also included sub-questions (probing questions) to further probe for more detailed or “richer” responses from the participants, where required. The first three interviews were considered pilot interviews during which the researcher observed whether the questions were understood by participants and provided the information required. These interview questions were consequently deemed appropriate after the initial interviews, and were continued with:

Question 1: Tell me about your personal well-being.

- How are you currently doing?
- How do you feel?
- Are you experiencing any concerns regarding your physical health?
- Are you experiencing any concerns regarding your psychological health?

Question 2: What factors contributed to your current state of well-being?

- What impact did this have/is this having on you?
- Are there things happening in your personal/family life that are contributing to the stress and/or exhaustion you are experiencing?
- Are there things happening in your work life that are contributing to the stress and/or exhaustion you are experiencing?

During the in-depth interviews the researcher captured detailed notes of the experiences and perspectives shared by participants and input these notes on a secure (password-protected) electronic (Microsoft Excel) spreadsheet. The researcher was conscious of the sensitive nature of the interviews and was guided by previous experience with such interviews in the organization, which led her to not record the participants’ interview, as participants generally feel more comfortable without their voices being recorded. At the end of each interview, the researcher confirmed that the notes captured were an accurate reflection of what the participant meant when answering the questions. This was done by sharing the notes captured during the discussion with participants via e-mail; the e-mail served both to confirm the accuracy of the notes and to re-emphasize the availability of further support if needed by the participant.

### Data analysis

A thematic analysis approach was followed to assist the researcher with identifying, analysing, and reporting themes within the data (Braun & Clarke, 2006). The following six phases of thematic analysis as identified by Braun and Clarke (2006) guided the researcher in the execution of this study: (1) familiarization with the data, (2) coding, (3) searching for themes, (4) reviewing themes, (5) defining and naming themes, and (6) writing up.

#### Step 1: Familiarization with the data

The researcher immersed herself in the data by allowing sufficient time for reading and re-reading the text. This allowed the researcher to start identifying and noting potentially interesting features of the data that were relevant to the research question (Clarke & Braun, 2014) and helped her to go back to them in later phases.

#### Step 2: Coding

Once the researcher had familiarized herself with the data she started the process of coding, which involved working through the text, line-by-line, as a process to identify units of meaning, and labelling these with a code that captured the meaning identified (Willig, 2013). The researcher, together with two co-coders (from the same field of study), went through a process of manually coding the data to ensure an accurate and enhanced process of identification of codes.

#### Step 3: Searching for themes

In this phase, the researcher and co-coders actively worked towards constructing themes by analysing the identified codes to ascertain how the different codes could be clustered together to form overarching themes (Braun & Clarke, 2006). Themes were identified by reviewing responses from participants pertaining to three identified categories (job demands, life demands, and health concerns); sub-themes, and keywords describing sub-themes, were also captured in a separate document as the researcher proceeded through the data analysis.

#### Step 4: Reviewing themes

The researcher spent time ensuring that each theme told a compelling story about the data, and worked on defining the boundaries of each individual theme (Braun & Clarke, 2006; Clarke & Braun, 2014). Where it was required, the researcher also collapsed and split themes, and discarded themes for being insignificant.

#### Step 5: Defining and naming themes

The aim of this analytical process of refinement was to produce a detailed definition of each theme, which captured its shape and texture and showed how it relates to other themes (Clarke & Braun, 2014). Sub-themes were also refined during this phase and descriptive keywords for the sub-themes were finalized.

#### Step 6: Writing up

During the last phase of the thematic analysis, the researcher wrote up the data in such a way as to clearly and accurately present her findings of the data. By combining analytical narrative and data abstraction, the researcher aimed to contextualize the findings and also to reinforce the validity of the interpretations (Clarke & Braun, 2014). It is important to keep in mind that all of the co-coders were licensed industrial and organizational psychologists registered with a statutory health professions council.

### Strategies to ensure quality data

For the purposes of this study, the four constructs of trustworthiness as proposed by Guba (1981) were considered, i.e., credibility, transferability, dependability, and confirmability.

### Credibility

Credibility is the alternative to internal validity in quantitative terms (De Vos et al., 2005). The researcher strived to ensure that participants’ views and responses to the phenomenon being studied were accurately captured and presented in a meaningful way with the aim of establishing credibility (Shenton, 2004; Thomas & Magilvy, 2011). The researcher managed to establish a truthful account of the perspectives of demands as experienced by participants through sharing the notes captured with participants and confirming the accuracy and truthful representation thereof.

### Transferability

Transferability is established through comprehensively describing the context and setting within which the research was conducted, to allow the reader to determine the extent to which the case could be applied to other situations and populations (Shenton, 2004). To achieve this outcome, the researcher took great care to meticulously describe the research process, context, and participants in the present study. This contributed towards creating a clear picture of the context and setting of this study for the reader.

### Dependability

Dependability aims to create an audit trail that enables future researchers to repeat the study (Thomas & Magilvy, 2011). The researcher took care to present the study in such a manner that it allows the reader to see and understand the research process followed to make decisions and interpretations. This was done through meticulous reporting of the processes within the study as well as of the research methods used. By doing this, the researcher was able to vividly present the research process and methodology followed in this study to create an audit trail which can be used by future researchers to repeat the study.

### Confirmability

Confirmability is similar to objectivity in quantitative terms, and requires the qualitative research to be reflective and to maintain a sense of awareness and openness to the study and unfolding results (Thomas & Magilvy, 2011). The researcher made a conscious effort to let participants lead the direction of the interview and to follow by asking clarifying questions, where required (Thomas & Magilvy, 2011). While conducting this study, every effort was made by the researcher to keep an open mind as to how the results were unfolding and not to let her personal values and biases affect the research.

### Ethical considerations

To ensure that the research project was handled in an ethical manner, and that the dignity, rights, and well-being of participants were considered, the researcher was guided by the following principles.

#### Avoidance of harm

The researcher took care to ensure that no participants were harmed during the research process.

#### Confidentiality and privacy

Care was taken by the researcher to safeguard the privacy and identity of participants. All collected information was handled in a confidential manner and unique numerical identifiers were used to ensure the anonymity of all participants.

#### Informed consent

The researcher obtained informed consent from both the participants and the organization where the data were collected, with the commitment that all data would be treated in a confidential manner.

#### Voluntary participation

In positioning the project to employees in the organization, it was clearly communicated that involvement in the project was voluntary and free from any coercion. Participants could therefore decide whether they wanted to participate in the project, or not, and could end their involvement in the project at any point. As a result, only completed surveys could be included in the study and incomplete surveys had to be discarded. Where individuals gave permission for the organizational development team to view their results, individuals at risk of burnout were invited to participate in an interview.

#### Security of data

Data collected for the study were kept confidential in a secure managed data warehouse that is password protected.

## Results

The findings of the study were structured into categories, themes, and sub-themes. The three categories identified for structuring of the data were job demands, life demands, and health concerns. Tables containing themes, sub-themes, and descriptive keywords were used to present the data within each category.

### Category 1: Job demands

Participants were asked to describe factors which contributed to their current state of well-being, and through the process of data analysis “job demands” was identified as a category (Table I). It was evident from the results that most of the participants experienced job demands as a factor impacting upon their well-being. All responses related to job demands were grouped together in this category.

**Table I.** Job demands.

Theme	Sub-theme	Descriptive keywords
Change management	Change of manager	Change of manager in team
	Lack of communication	Does not get communicated properly and positioned on why changes are made
	Leadership	Leadership changes in area
	Uncertainty	Why are these changes being made?
Cognitive demands	Mental load	Too many things to remember; very busy with a lot of things; working with a lot of detail and doing the same things over and over
Emotional demands	Emotion regulation	Gets worked up about things that should not affect one; internalizes when things go wrong that is not one’s fault and beats oneself up over it
	High emotional load	Working in a high emotional load environment; emotional load is high; emotionally drained
	Difficult clients	Clients are often irate and shout over the phone
Job dissatisfaction	Unhappiness in role	Unhappy in one’s role
Job expectations	Not delivering	Ability to cope and manage the same output; output is lower
	Training team members	Readying a team member to second in charge and worried that not enough was done enough to prepare that team member
	Role ambiguity	Challenges and frustrations with having two managers in the department with different expectations and ways of working
	Role change	Moving into a new role; experienced some stress in role moving from one department to another, and had some uncertainty and feeling a bit out of one’s depth
	Role conflict	Conflicting demands
	Role overload	On top of work pressures: pay for performance, sales targets, systems, etc.
	Person–job (mis)fit	The picture that was painted when being recruited and how it unfolded were vastly different; disappointed that the role did not turn out to be what was expected
	Overcommitment	Overcommitted because does not want to let the business down
Job insecurity	Quantitative job insecurity	Does not feel secure in job, constantly working in fear of losing job
Remuneration	Pay for performance (PFP)	A lot of PFP changes made in department which negatively impacts salaries; uncertainty around PFP and not knowing what one will earn each month; finances also affected by changes made in department as they directly affect one’s salary; business case for change in PFP made but has a significant impact on one’s salary; concerned about how much one will earn to support oneself and one’s family when one goes on to PFP; PFP model does not reward one and it has a negative impact; uncertainty related to one’s remuneration on the work front
Work overload	Long hours	Working long hours; working so hard (most days 6 am–6 pm); working extremely long hours, caught in belief that no other option than to do that to try and get everything done; but admittedly comes at a cost in that one is here every day at 5:30 am, leaves late and is also here at the weekends; put in a lot of extra hours to find out how everything works; workload is very high and works until 11/12 every night; works longer; have to work late every day to meet targets and perform; perceived workload to be very high
	High workload	Swamped at work; high volume of claims to deal with; workload is very high; often feels overloaded; a lot happening in the workspace from a quantity perspective; works longer and harder
	Workaholism	Placing a lot of pressure on oneself to outperform; always available; feels guilty when off sick
Work–life balance	Work–family conflict	Work impacts on one’s family; unable to set boundaries in terms of work
	Family–work conflict	Bring family to work on weekends in order to get work done

Themes that were identified in this category included change management, cognitive demands, emotional demands, job dissatisfaction, job expectations, job insecurity, remuneration, work overload, and work–life balance.

#### Change management

A lack of communication and uncertainty around the reasons for the changes being made contributed to job demands experienced by participants. Change in leadership and change of manager also added to the job demands that participants experienced.

#### Cognitive demands

Participants identified the number of things they needed to remember as well as being very busy as factors adding to their mental load; the experience of having too many things to remember added to the cognitive demands experienced by participants.

#### Emotional demands

The experience of participants having to regulate their emotions as a result of getting worked up about things that should not affect them was another of the job demands identified by participants. They also identified having to deal with difficult clients who were irate and shouted over the phone, as well as having to deal with a high emotional load, as aspects which contributed to job demands.

#### Job dissatisfaction

Unhappiness in one’s role was identified as one of the factors that contributed to the level of job demands experienced by participants.

#### Job expectations

Participants named job expectations as adding to the demands experienced in their jobs. These included the experience of role ambiguity, role change, role conflict, role overload, person–job (mis)fit, overcommitment, and having to train team members. This theme also included not being able to deliver on expectations.

#### Job insecurity

Feelings of insecurity around the job (constantly working in fear of losing one’s job) were identified as a theme contributing to job demands.

#### Remuneration

Participants identified the pay for performance (PFP) remuneration structure as impacting on job demands. Included in this theme were changes to the PFP model that negatively impacted salaries, uncertainty around what one would earn each month, and whether this would be enough to support oneself and one’s family.

#### Work overload

Participants mentioned working long hours and dealing with high workload as factors contributing to job demands. Workaholism was also identified, which included feelings of always having to be available, and feeling guilty when off sick.

#### Work–life balance

Job demands were experienced by participants in having to deal with work–family conflict and family–work conflict.

### Category 2: Life demands

Participants not only experienced job demands, but also highlighted life demands in describing factors which contributed to their state of well-being. “Life demands” was consequently identified as a second category in which all responses relating to life demands experienced by participants were grouped together (Table II).

**Table II.** Life demands.

Theme	Sub-theme	Descriptive notes
Family demands	Death	Dad passed away
	Dependent on family	To gain independence from family
	Illness of family member	Mother has been in and out of hospital over last couple of months, not sure what reason is for the illness
	Parenting	Small baby of under a year; raising kids from two families; different parenting styles is a challenge; feel extremely stressed every second weekend when having all of the kids; stresses about December holiday coming up—going away with all the kids to visit family; also a new father—daughter is 8 months old
	Pregnancy	34 weeks of pregnancy and has been having a difficult time; had a few miscarriages; 8 months pregnant with second baby after 10 years and is physically a difficult pregnancy
	Relationship	Divorced from wife; relationship with fiancé ended; experiences challenges in marriage; having an impact on marriage; focus on creating a connection between father–daughter which is strained
	Retrenchment (redundancy)	Husband is facing possible retrenchment
	Support structures	Had to be strong for mother and sister; from an emotional and financial perspective mother relies on a lot of support (not really other family members that can support); only person working and need to look after family (send money home and brother at university)
	Work-to-family conflict	Have to bring family to work on weekends to get work done
Financial demands	Debt	Currently under debt review
	Expenses	Feels like things are spinning out of control, not able to pay all the bills; still need to pay own bills
	Medical expenses	High medical bills
	Relocation	Financial stressors around moving house and relocation
Health	Surgery	Went in for a knee (leg) operation
	Broken bones	Broke ankle
	Health scare	Had to deal with the possibility of having cancer
	Hernia; sleeping problems	Sleeping problems due to hernia
	Bipolar disorder	Diagnosed as bipolar and on medication
	Attention-deficit/hyperactivity disorder (ADHD)	Diagnosed with ADHD
	Depressive disorder	Using antidepressants for years
Personal demands	House hunting	Battling to find a house
	Studying	Started studying again after years

#### Family demands

Participants experienced life demands in having to deal with various family-related demands. Illness of a family member and death of a family member were identified as types of family demands. This was further experienced in matters of pregnancy and parenting, as well as work–family conflict, where it was necessary to bring family to work over the weekend to get work done. Participants were also faced with challenges in relationships and retrenchment (redundancy) of family members. Being dependent on family (and wanting independence) as well as having the responsibility of supporting the family added to the demands experienced by participants.

#### Financial demands

The financial demands on participants added to the life demands experienced. Financial demands included expenses (not being able to pay bills), including medical expenses, debt, and financial stressors around relocation.

#### Personal demands

Personal demands such as house hunting and studying were identified as life demands experienced by participants.

### Category 3: Health concerns

Participants were asked to share information about their personal well-being, including concerns regarding their physical and psychological health, to aid in gaining a comprehensive understanding of participants’ state of well-being at the time of the study (Table III). The following themes were extracted from the data: physical health and psychological distress.

**Table III.** Health concerns.

Theme	Sub-theme	Descriptive notes
Physical health	Surgery	Went in for a knee (leg) operation
	Broken bones	Broke ankle
	Health scare	Had to deal with the possibility of having cancer
	Hernia; sleeping problems	Sleeping problems due to hernia
	Bipolar disorder	Diagnosed as bipolar and on medication
	Attention-deficit/hyperactivity disorder (ADHD)	Diagnosed with ADHD
	Depressive disorder	Using antidepressants for years
Psychological distress	Anxiety	High anxiety; anxious because he is alone at home; increasing anxiety levels
	Breakdown	On the verge of a breakdown; world “comes crashing down”
	Cognitive ability (impaired)	Impact on cognitive ability due to hernia and pregnancy
	Cynicism	Sees everything negatively; has a “whatever” attitude
	Despondency	Behaving differently because of despondency
	Exhaustion (mental)	Mentally tired; tired with work in general
	Forgetful	Forgets important things; forgets small things
	Flustered	When failing to meet standards gets completely flustered; gets flustered
	Frustration	Frustrated at how communication happens; feeling frustrated at colleagues; frustrated with lack of career progress
	Guilt	Feeling guilty due to not being at work; felt guilty for being sick; feeling bad due to delegating work
	Hates work	Hates coming to work—takes 30 min to walk from the basement to desk
	Irritability	Makes errors and is more irritable; irritated
	Overwhelmed	Feeling overwhelmed; holding too many ropes together in life
	Preoccupied; stagnant	Preoccupied and feels stuck
	Self-inflicted pressure	Self-inflicted pressure to try to deliver at the same standard
	Self-talk (negative)	Self-talk is negative and not used to this
	Shock; disappointment	Shocked and disappointed when called into a meeting to be told of negative influence

#### Physical health

Participants identified various factors related to their physical health. These included having a health scare, as well as surgery, broken bones, hernia (which contributed to sleep problems), bipolar disorder, depressive disorders, and attention-deficit/hyperactivity disorder (ADHD).

#### Psychological distress

Participants experienced psychological distress which related to health concerns. This referred to situations where participants experienced feelings of anxiety, cynicism, despondency, exhaustion (mental), frustration, guilt, irritability, and being forgetful and flustered. Participants also shared feeling overwhelmed, on the verge of a breakdown, and hating work. Other aspects that were highlighted by participants as health concerns included (impaired) cognitive ability, and feeling preoccupied and stagnant.

## Discussion

The general objective of the study was to explore demands from the perspective of employees identified as being at risk of burnout. Responses from the participants provided strong evidence for the notion that the experience of burnout was influenced by both job demands and life demands. Participants also highlighted health concerns experienced, which included concerns around both physical health and psychological distress in sharing their experiences of demands. The researchers will now discuss the three categories that emerged from the research results.

### Job demands

Strong empirical evidence exists in the literature to support the view that excessive job demands are a key predictor of burnout (Alarcon, 2011; Bakker, Demerouti, & Verbeke, 2004; De Beer et al., 2016; Demerouti, Bakker, Nachreiner, & Schaufeli, 2001). Participants viewed change management as a form of job demand and experienced it through a lack of communication, uncertainty about why changes were made, and changes in leadership. According to Rumbles and Rees (2013), organizations tend to communicate change badly, and do very little to protect the well-being of employees or to manage stress in times of change.

Cognitive demands was also identified as a theme that contributed to the experience of job demands. Participants experienced cognitive demands in the number of things they had to remember, which subsequently added to a high mental load. According to a study by Nahrgang, Morgeson, and Hofmann (2011), the overall complexity of work (aspects of the job that require mental effort such as cognitive demands, task complexity, and ambiguity) is the second largest predictor of burnout. In the operational and support areas of the organization where the study was conducted, the reality was that employees were often expected to deal with more than one matter at the same time. Certain roles (e.g., actuarial and finance positions) also required employees to deal with high levels of complexity, and expectations of what needed to be delivered within certain roles were subject to change in alignment with changes to business priorities. This made a higher cognitive load likely.

The findings of the present study showed emotional demands to be the third theme extracted from the data under the category of job demands. Castanheira and Chambel (2013) support this finding by providing evidence for the positive relationship between emotional dissonance (the requirement to display unfelt emotion) and burnout. Participants in this study highlighted the need to regulate emotions and “not getting worked up about things that should not affect one” as one of the factors contributing towards experiencing emotional demands. Not being cognizant of this stressor and not providing employees with coping skills or with the opportunity to debrief from high emotional demands can cause a risk to the well-being of employees, specifically in occupations such as call centres, where dealing directly with customers is a daily requirement of the job. Lings, Durden, Lee, and Cadogan (2014) also confirmed that different socio-emotional demand constructs act to increase burnout symptoms when displayed emotions are incongruent with felt emotions or with service norms.

Job expectations, job dissatisfaction, and job insecurity all emerged as themes within the job demands category. Of these, job expectations was the theme occurring most often and having various sub-themes feeding into it. A study by Faúndez et al. (2014) confirmed the impact of role ambiguity and role conflict on burnout; both were identified as sub-themes under job expectations by participants in the present study. Anand, Nagle, Misra, and Dangi (2013) found organizational role stress (including role expectation conflict, role overload, and personal inadequacy) to be significantly related to depersonalization and emotional exhaustion dimensions of burnout. Job dissatisfaction as a theme reflected the unhappiness of employees in their current roles. Previous research has confirmed an association between staff burnout and outcomes such as job satisfaction and intention to leave the organization (Skirrow & Hatton, 2007). Job insecurity spoke to employees who did not feel secure in their jobs, and could potentially also lead to employees exiting the organization in pursuit of greater job security and stability. Bosman, Rothmann, and Buitendach (2005) and Kozak, Kersten, Schillmöller, and Nienhaus (2013) support the significant impact of job insecurity on personal burnout.

Another finding of the study was the impact of remuneration, specifically PFP, as a demand experienced by participants. Participants highlighted changes made in the remuneration model, as well as the uncertainty around how much one would earn, as elements contributing to the stress that they experienced. A study by Nieuwenhuijsen, Bruinvels, and Frings-Dresen (2010) found that a high effort–reward imbalance predicted the incidence of stress-related disorders. The central principle of the effort–reward imbalance model is that an imbalance between (high) efforts and (low) rewards leads to (sustained) strain reactions (Van Vegchel, De Jonge, Bosma, & Schaufeli, 2005).

Participants also experienced work overload (long hours, high workload, and workaholism) as a theme contributing to demands. This result is in alignment with previous studies which found work overload to predict burnout (Bakker & Demerouti, 2007; De Beer et al., 2016). The reality of high workloads and the associated risks causes various challenges for organizations as the current economic climate necessitates a lot of organizations to expect employees to deliver greater outputs with fewer resources (Evenstad, 2015) to ensure the profitability and sustainability of the business.

Work overload led into the next theme identified by participants, namely work–life balance, which included work–family conflict and family–work conflict. Karatepe (2013) also found that employees who have high workloads and are unable to establish a balance between work (family) and family (work) roles are emotionally exhausted. Various research studies have been conducted around this topic, investigating the impact of work–family conflict on burnout (Hall, Dollard, Tuckey, Winefield, & Thompson, 2010) and confirming the positive association of family on work conflict with burnout (Lambert, Hogan, & Altheimer, 2010).

### Life demands

The results indicated that participants experienced not only job demands, but also life demands. The literature supports the finding that burnout is associated with non-occupational factors such as personal and stressful life events (Dyrbye et al., 2006; Peeters et al., 2005; Schonfeld & Bianchi, 2016). Participants cited family demands, financial demands, and personal demands as themes contributing to the experience of life demands.

Family demands included sub-themes of death, dependence on family, illness of a family member, parenting demands, being pregnant, relationship challenges, retrenchment of a family member, support structures, and work–family conflict. In a study by Dyrbye et al. (2006) which confirmed the impact of personal life events on professional burnout, the number of “negative” personal life events experienced (e.g., divorce, illness of a close family member, and death of a family member) were specifically highlighted.

Financial demands referred to in this study included matters of debt, expenses (the inability to pay bills), medical expenses, and financial stressors around moving house and relocation. A study by Starrin, Åslund, and Nilsson (2009) confirmed that the greater the financial stress and the more experiences of having been shamed, the greater the risk of psychosocial ill-health (anxiety, depression, and reduced psychological well-being). Considering the impact of financial stress (together with shaming) on the well-being of employees, it becomes critical for organizations to look at ways in which to support employees in managing their finances and achieving financial wellness.

Personal demands experienced by participants in this study included house hunting and studying. Moving into a new house (Raviv, Keinan, Abazon, & Raviv, 1990) and studying (Robotham & Julian, 2006) have been shown in the literature to be sources of stress.

Specific references to the relationship between financial demands and burnout, as well as between personal demands (house hunting and studying) and burnout, could not be found in the literature, and could be recommended as potential focus areas for future studies.

### Health concerns

This category contributed to creating a thorough understanding of the experiences of well-being from the perspective of participants. Two themes were identified within the health concerns category, namely physical health and psychological distress.

Physical health included sub-themes such as a health scare, surgery, broken bones, and sleeping problems, as well as bipolar disorder, depressive disorder, and ADHD. Various studies have examined the relationship between burnout and depression and have yielded mixed results on whether burnout should be viewed as a distinct phenomenon or as a symptom of depression (Bianchi et al., 2015; Thuysma & De Beer, 2016). Burnout has been reported to predict depressive symptoms, and Thuynsma and De Beer (2016) found that depressive symptoms (together with job demands and satisfaction with life) explained significant amounts of variance in the burnout construct. A study by De Beer, Pienaar, and Rothmann (2014) also found a positive relationship between burnout and self-reported treatment for health conditions, with the relationship with treatment for depression being the strongest. Therefore, being aware of the close relationship between burnout and depression will empower individuals with the knowledge to seek professional help more quickly and receive the correct treatment.

Burnout has been demonstrated to lead to other physical health concerns such as sleep disturbances, headaches, respiratory infections, and gastrointestinal infections (Kim et al., 2011). Although the focus in the literature was mainly on the impact of burnout on the health of individuals (as a result of the health-impairment process), it may be necessary to also consider that existing health-related concerns such as indicated in the current study (e.g., health scares and surgery) could play a contributory role in the development of burnout. The literature provided evidence for this downward spiralling effect on employee well-being (e.g., Bowen, 2013).

The psychological distress theme included multiple sub-themes ranging from anxiety, frustration, irritability, and guilt, to cynicism, despondency, and negative self-talk. Previous research studies provided support for the notion that burnout plays a role in the development of psychological distress (Kozak et al., 2013). A study by Sánchez-Moreno, Roldán, Gallardo-Peralta, and de Roda (2015) showed a strong association between burnout and psychological distress, and confirmed that it is the emotional exhaustion dimension of burnout that shows the strongest correlations with lack of health or well-being in the particular study. The study by Sánchez-Moreno et al. (2015) was conducted with social workers and the aforementioned results therefore indicate the fundamental role of emotions within professions where managing emotions forms an integral part of daily professional life (e.g., social work). This highlights an important consequence for the current study, as various roles within the operational areas e.g., call centre advisers, required participants to manage their emotions as an integral part of conducting their daily work. Sánchez-Moreno et al. (2015) confirmed the importance of informal social support as a variable negatively related to distress.

### Limitations and recommendations

The present study was not without limitations. It is important to emphasize that the findings of this study were related to employees in a financial services organization in South Africa, and it cannot be assumed that they would be applicable to all other settings and organizations.

Another potential limitation could be that participants may have felt hesitant to share certain information owing to the sensitive nature of the topic being discussed. However, the interviewer felt comfortable that a satisfactory level of safety, to allow open and honest feedback to be shared by participants, was in place.

For future research, it is recommended that the study is supported by the collection of quantitative data. A longitudinal study exploring demands from the perspective of employees identified as being at risk of burnout over a period of time will also add to an enhanced understanding of changes in the experience of burnout over time and factors contributing to the experiences of burnout over time.

It is also recommended that future research focuses on exploring ways in which organizations can provide support to employees to assist them in better dealing with the demands experienced, and provide employees with training to better equip them in dealing with challenges faced in work and life. This could include training to empower employees with skills to effectively raise concerns around demands being experienced in the workplace, and help employees to explore alternative ways of achieving the desired results through open and candid dialogue. Other training interventions could potentially include stress management and conflict management.

A further recommendation would be for future studies to explore areas of positive deviance. This could involve investigating areas in the business where there is a lower incidence of employees being at risk of burnout and exploring the factors present in the environment that could contribute towards this outcome, e.g., increased work engagement.

## Conclusion

It is evident that the experience of demands from the perspective of employees at risk of burnout falls within the domains of both work and life. The results of this study provide a reflection of both physical and psychological health concerns experienced by employees at risk of burnout. Research increasingly indicates that not considering the management of well-being of employees as a key determinant to the success of a business could be to the detriment of organizations. Organizations and relevant professionals should therefore be encouraged to consider the findings of the current study in determining the most suitable manner in which to provide ongoing support to their employees.
